# Integrated bioinformatical and *in vitro* study on drug targets for liver cirrhosis based on unsupervised consensus clustering and immune cell infiltration

**DOI:** 10.3389/fphar.2022.909668

**Published:** 2023-01-04

**Authors:** Qingjia Chi, Di Wang, Ting Sun, Hua-Ping Liang

**Affiliations:** ^1^ Department of Engineering Structure and Mechanics, School of Science, Wuhan University of Technology, Wuhan, China; ^2^ State Key Laboratory of Trauma, Burns and Combined Injury, Department of Wound Infection and Drug, Research Institute of Surgery, Daping Hospital, Army Medical University, Chongqing, China; ^3^ School of Life Science and Engineering, Southwest Jiaotong University, Chengdu, Sichuan, China; ^4^ Surgical Laboratory, General Hospital of Ningxia Medical University,, Yinchuan, Ningxia, China

**Keywords:** liver cirrhosis, gene expression, prognostic value, immune microenvironment, unsupervised clustering

## Abstract

Liver cirrhosis is one of the most common cause of death in the world. The progress of liver cirrhosis involves health, liver cirrhosis and liver cancer, leading to great challenges in the diagnosis of the disease. Drug targets, which could be obtained conveniently, can help clinicians improve prognosis and treatment. Liver cirrhosis is associated with serum calcium levels. And studies reported Tanshinone IIA plays a therapeutic role in liver injury through activating calcium-dependent apoptosis. In this study, we explored the diagnostic key targets of Tanshinone IIA in liver cirrhosis through exploration of comprehensive dataset including health, liver cirrhosis and liver cancer patients. The unsupervised consensus clustering algorithm identified 3 novel subtypes in which differentially expressed genes (DEGs) between both subtypes were found by pairwise comparison. Then, 4 key drug targets of Tanshinone IIA were determined through the intersection of these DEGs. The diagnostic performance of target genes was assessed and further verified in the external dataset. We found that the 4 key drug targets could be used as effective diagnostic biomarkers. Then the immune scores in the high and low expression groups of target genes were estimated to identify significantly expressed immune cells. In addition, the immune infiltration of high and low target gene expression groups in several immune cells were significantly different. The findings suggest that 4 key drug targets may be a simple and useful diagnostic tool for predicting patients with cirrhosis. We further studied the carcinogenesis role of AKR1C3 and TPX2 *in vitro*. Both mRNA and protein expression in hepatoma carcinoma cells was detected using qRT-PCR and Western blot. And the knockdown of AKR1C3 and TPX2 significantly suppressed cell proliferation, migration and invasion.

## Introduction

Liver cirrhosis is a worrisome medical condition worldwide (Duah and Nkrumah, 2019). It is a sequelae of chronic liver diseases and is characterized by replacing liver tissue with fibrosis, scar tissue, and regenerative nodules (Wong, 2016). Cirrhosis can remain compensated for many years before a decompensation event occurs. Decompensated cirrhosis is an end-stage liver disease characterized by developing complications, including jaundice, variceal bleeding, ascites, and encephalopathy, and significantly reduced survival (EAF Of liver, 2018; Gines et al., 2021).

Common causes of liver cirrhosis are infection with hepatitis virus and alcohol-related liver disease. Liver cirrhosis is the 11th most common cause of death in the world. About 2 million people die from liver cirrhosis each year. In China, patients with cirrhosis account for 20% of the total patients with liver chronic. About 50% of the world’s liver cancer death and 15% of liver cirrhosis occur in China (Tan et al., 2022). In China, more than 80% of patients developed HCC (Tsai et al., 2020) in the context of liver cirrhosis. There is no effective treatment for liver cirrhosis. Treatment is mainly concentrated on the cause and symptoms, which can only relieve the disease. It is difficult to predict the development and prognosis of liver cirrhosis. The uncertainty of the disease is likely to cause the deterioration of the medical behavior of the patient and the interruption of the disease treatment (Wu et al., 2022).

The 1-year mortality rate of compensated liver cirrhosis is 1%–3.4%, but the mortality rate after compensatory decompensation increases to 20%–57% (D'Amico et al., 2006). The high mortality of late liver cirrhosis highlights the necessity of prognostic improvement. Therefore, identifying diagnostic biomarkers and exploring reliable drug targets can guide clinical treatment. Liver cirrhosis is associated with serum calcium levels (Bandi et al., 1997; Kim et al., 2021). Platelet calcium ion depth was significantly lower in cirrhotic patients than in normal controls. Tanshinone IIA induces an increase in intracellular calcium and lead to increased mobilization (Yang et al., 2005; Fan et al., 2011). And studies reported Tanshinone IIA plays a therapeutic role in liver injury through activating calcium-dependent apoptosis (Dai et al., 2012). The drug targets can be obtained through simple, non-invasive, and repeated ways. Thus, key targets of Tanshinone IIA provided solutions for diagnosing liver cirrhosis and therapy intervention.

We previously used machine learning algorithms to examine the role of immune infiltration in various diseases (Xu et al., 2022; Zhao et al., 2022). In this paper, we have determined the clinical and prognosis of patients with cirrhosis and discussed their potential applications in the future. We generated three groups with significant differences through the unsupervised consensus cluster algorithm. The volcanic plot selected Differentially expressed genes (DEG) in these three groups. Then we used the Venn plot to determine the four key drug targets of Tanshinone IIA. Use the violin diagram to evaluate the diagnostic value of the target gene. The scores of 22 immune cells in targets are high-expression and low-expression, and they have identified significantly different cells through. We further studied the carcinogenesis role of several key targets *in vitro*.

## Materials and methods

### Microarray data

Microarray data were downloaded from the Gene Expression Omnibus (GEO) (2022.4.1) database GSE54238 based on platform GPL16955 (26 HCC samples, 228 cirrhotic samples, and 10 adjacent non-cancerous samples) and GSE63898 (228 HCC samples and 168 cirrhotic samples) based on platform GPL13667. The data obtained by the GEO data access policy is publicly available and open access.

### Unsupervised clustering

We download the “ConsensusClusterPlus” R package to perform unsupervised consensus clustering. The algorithm is based on the computational method, namely consensus clustering. It allowed cases to be divided or compressed into multiple distinct clusters based on provided flags or signatures clusters. Furthermore, landmark gene sets summarize and represent specific well-defined biological states or processes and show consistent expression. Based on Molecular Signature Database.

### Functional enrichment analysis

DAVID was used to perform functional and pathway enrichment analyses to assess the biological significance (Dennis et al., 2003; Xie et al., 2011). We used GOplot and ggalluvial respectively and R package for analysis.

### Differentially expressed gene (DEG) analysis

The R package “limma” was used to compare each other between Cluster 1, Cluster 2 and Cluster 3. And DEGs related to drug targets were identified (|log 2-fold change (FC)| > 1.0 and FDR < .05).

### Independent prognostic value of target genes

A violin plot was drawn to demonstrate the differential expression of target genes (HCC vs cirrhosis). We calculated the area under the AUC of the target gene.

### Relationship between target genes and immune cell infiltration

The CIBERSORT and ESTIMATE algorithms were performed. The ESTIMATE algorithm was used to determine the immune scores of all samples. The differences in immune cell infiltration between high and low target genes were analyzed based on the CIBERSORT algorithm.

### TIMER database analysis

TIMER is a comprehensive database that applies a deconvolution approach to assess immune infiltration (Li et al., 2016). We analyzed the correlation of the expression of four drug targets with immune infiltrate abundance in liver tissue.

### Cell lines and cultured medium

Human hepatocellular carcinoma cell line SNU423 was purchased from ATCC. The cells were cultured in RPMI 1640 supplemented with 10% FBS. Cell line was tested mycoplasma free using colorimetric mycoplasma detection assay with HEK-Blue-2 cells as described before.

### qRT-PCR and Western blot

Total RNAs were extracted by using RNeasy Kit from Qiagen following the manufacturer’s protocol. RNA concentration was measured by Nano Drop 2000. First strand of cDNA was synthesized by using PrimeScript RT Reagent Kit from TAKARA. qPCR was performed by using SYBR Green master mix purchased from Bio-rad company. Primers for beta-ACTIN (Forward: CAC​CAT​TGG​CAA​TGA​GCG​GTT​C, Reverse: AGG​TCT​TTG​CGG​ATG​TCC​ACG​T), Primers for AKR1C3 (forward: TCC​GAC​CAG​CCT​TGG​AAA​AC, reverse: TCT​GTT​GGT​GAA​AGT​TCC​TCA​C). Primers for TPX2 (Forward: 5′-ATA​TGT​GCC​CCT​TGC​ACA​GC-3′, reverse: 5′-ACA​GGA​GTC​TGT​GGG​TCT​CT-3′).

Total cellular protein were extracted with RIPA lysis buffer. Protein concerntration was diluted to 1 mg/ml with distilled water and lysis buffer, then heated in 95°C metal bath for 7 min. Electrophoresis was performed loading as 20 μg/well and proteins were transferred onto PVDF membranes using bio-rad semi-dry transfer machine. Membrane was blocked by 5% milk in PBST for 1 h at room temperature and them incubated with primary antibody for overnight. After washing out primary antibody, membrane was incubated with secondary antibody for 1 h and goes for imaging. A primary antibody against β-actin (1:3,000) was purchased from Santa Cruz Biotechnology. Antibody against AKR1C3 was purchased from Invitrogen (ARC0857). Antibody against TPX2 was purchased from Cell Signaling Technology.

### Cell proliferation, migration and invasion

Cells were seeded in 6-well plates and transfected with siRNAs, respectively. siRNAs targeted AKR1C3 or TPX2 were ordered from BGI Genomic. Sequences of siRNAs are listed below. AKR1C3 siRNA1: 5′-UUU​ACA​CAC​UGG​UGU​UUG​GAA-3′, siRNA2: 5′- AUC​AUU​UAG​CUU​UAC​ACA​CUG-3′; TPX2 siRNA1:5′- UUC​UUU​CUG​UUC​CAA​AUC​CUU-3′, TPX2 siRNA2: 5′-UUU​UUA​CAU​GAU​GCU​UUU​CUU-3′.

For cell proliferation, cells were seeded as 1 × 10^5^ per well in 6 well plates. After 72 h, cells were harvested and counted with cell counting chamber. For migration assay, cells were detached from tissue culture plate by using 0.25% Trypsin-EDTA solution and resuspended in serum free culture medium seeded as 5 × 10^5^ per well in a 6-well plate before scratching. 24 h later, cells were scratched with a 20 μl tip and washed twice with PBS and cultured in serum-free medium. Images were captured immediately after scratching, and then at 24 h. Nine measurements were performed for each group. Migration distances were measured by ImageJ software.

Cell invasion assay was performed using 8 µm Matrigel invasion chambers (Corning Company). Cells were detached after being transfected with siRNAs for 48 h. Then 2.5 × 10^4^ cells were seeded on top of the insert with serum-free medium. Lower chambers were filled with growth medium with 10% FBS. After 22 h, media containing remaining cells that did not migrate from the top of the membrane was carefully removed. Cells were fixed by putting the insert in formalin for 15 min, air-drying for 10 min at room temperature, and staining with 1% crystal violet solution for 10 min. The fixed inserts were then washed with distilled water. Three replicate experiments were performed. Trans-wells were imaged by microscope after staining with crystal violet. Five views were counted for each group.

### Statistical methods

Statistical data obtained from GEO were statistically analyzed by R (3.6.1) software (https://www.r-project.org/). The t-test was used between the two independent samples in the research, and *p* < .05 was considered statistically significant.

## Results

### Unsupervised consensus clustering identified three novel subgroups

**FIGURE 1:**
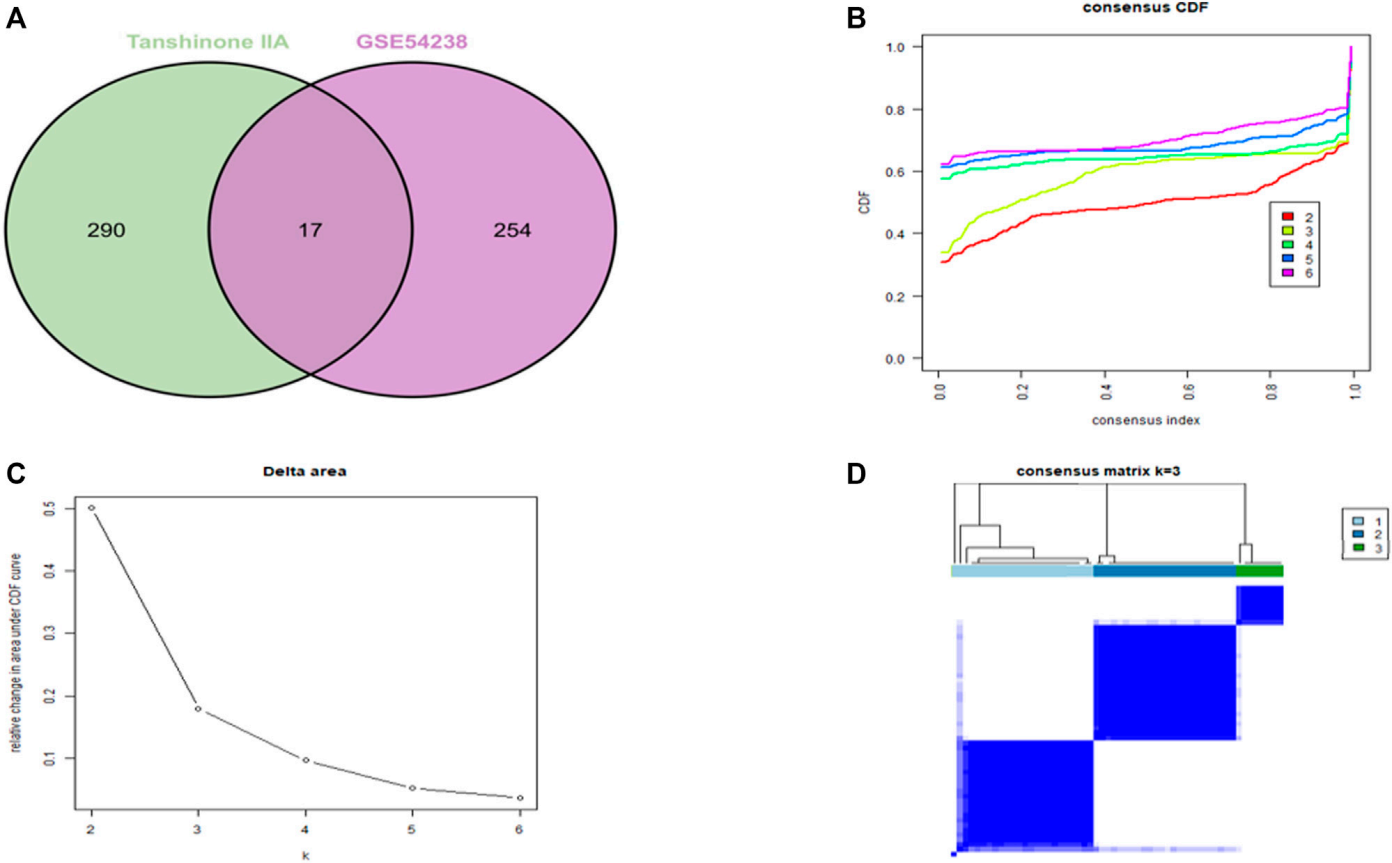
Selection of target genes. **(A)** Venn diagram for screening drug target genes. **(B)** CDF plot showing consensus distribution of target genes for each cluster (K). **(C)** Delta area plot, reflecting the relative change in the area under the CDF curve. **(D)** Consensus matrix heatmap depicting consensus values on a white to blue scale for each cluster.

We utilized intersections between drug targets of Tanshinone IIA and GSE54238 to select overlapping genes (Figure 1A). The CDF plot shows the consistency distribution for each cluster (Figure 1B). The elta area plot shows the relative change in the area under the CDF curve (Figure 1C). The largest change in area occurs between *k* = 2 and *k* = 4, where the relative increase in the area becomes significantly smaller. The mean cluster of the three clusters consensus scores were comparable (Figure 1D). Therefore, three novel subgroups that best represent data patterns in patients were identified using consensus clustering analysis.

### Functional pathway enrichment analysis

**FIGURE 2:**
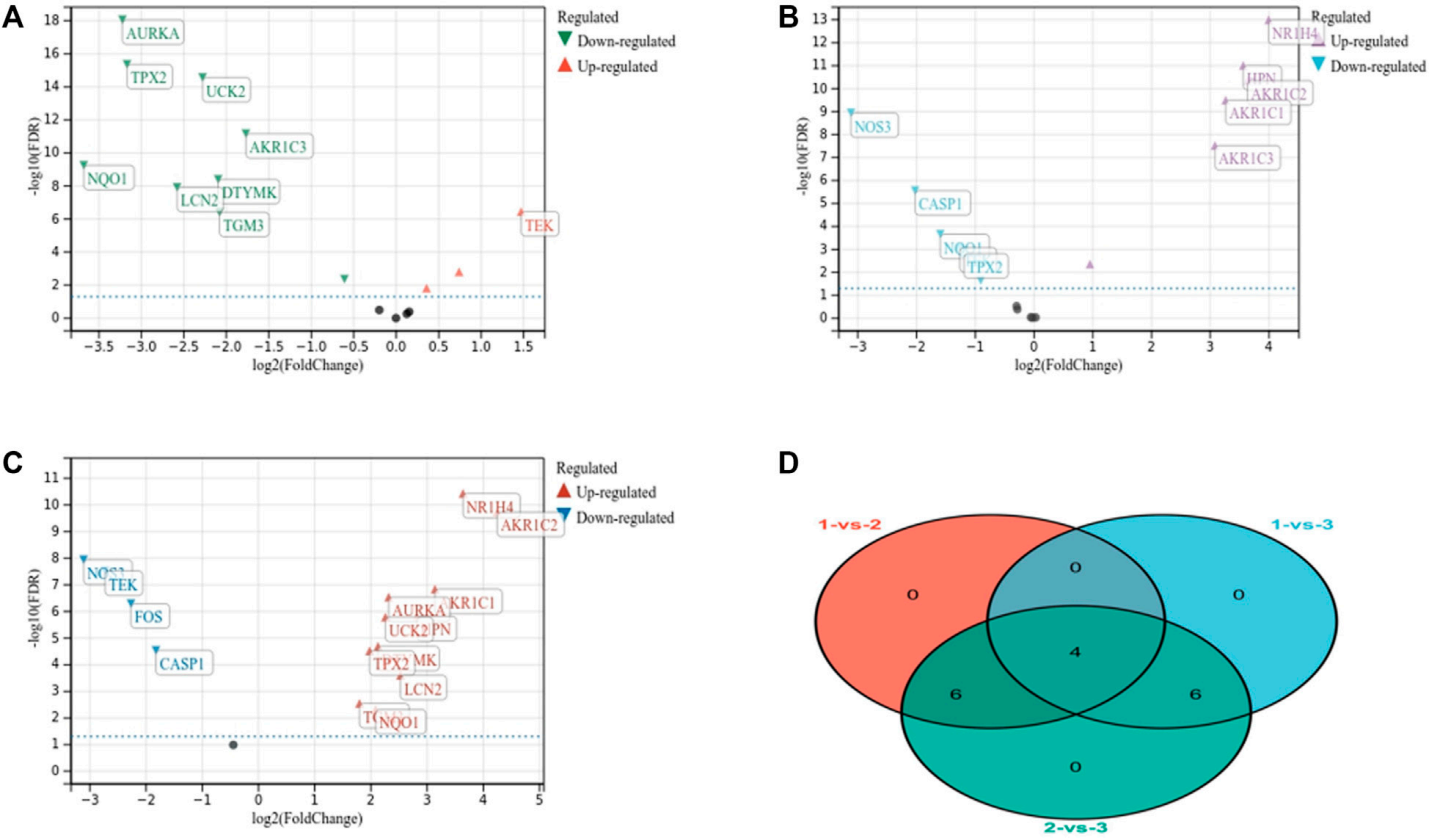
GO and KEGG enrichment analysis. **(A)** Biological process. **(B)** Cellular components. **(C)** Molecular function. **(D)** KEGG pathway.

We performed GO analysis on the intersected drug targets to reveal changes in biological process (BP), molecular function (MF) and cellular components (CC). The daunorubicin metabolism, doxorubicin metabolism, progesterone metabolism, prostaglandin metabolism, and the positive regulation of protein kinase B signaling were significantly enriched BP (Figure 2A). The drug target’s main CC was in the cytosol, axon terminals and cytoplasm (Figure 2B). These targets were mainly involved in MF including bile acid-binding, ketosteroid monooxygenase activity, steroid dehydrogenase activity, androsterone dehydrogenase activity, carboxylic acid-binding and the same protein binding (Figure 2C). Meanwhile, the drug targets were significantly enriched in the KEGG pathway, including steroid hormone biosynthesis, fluid shear stress, atherosclerosis-pyrimidine metabolism, ubiquinone, and other terpenoid quinone biosynthesis (Figure 2D).

### Identification of differentially expressed genes between clusters

**FIGURE 3:**
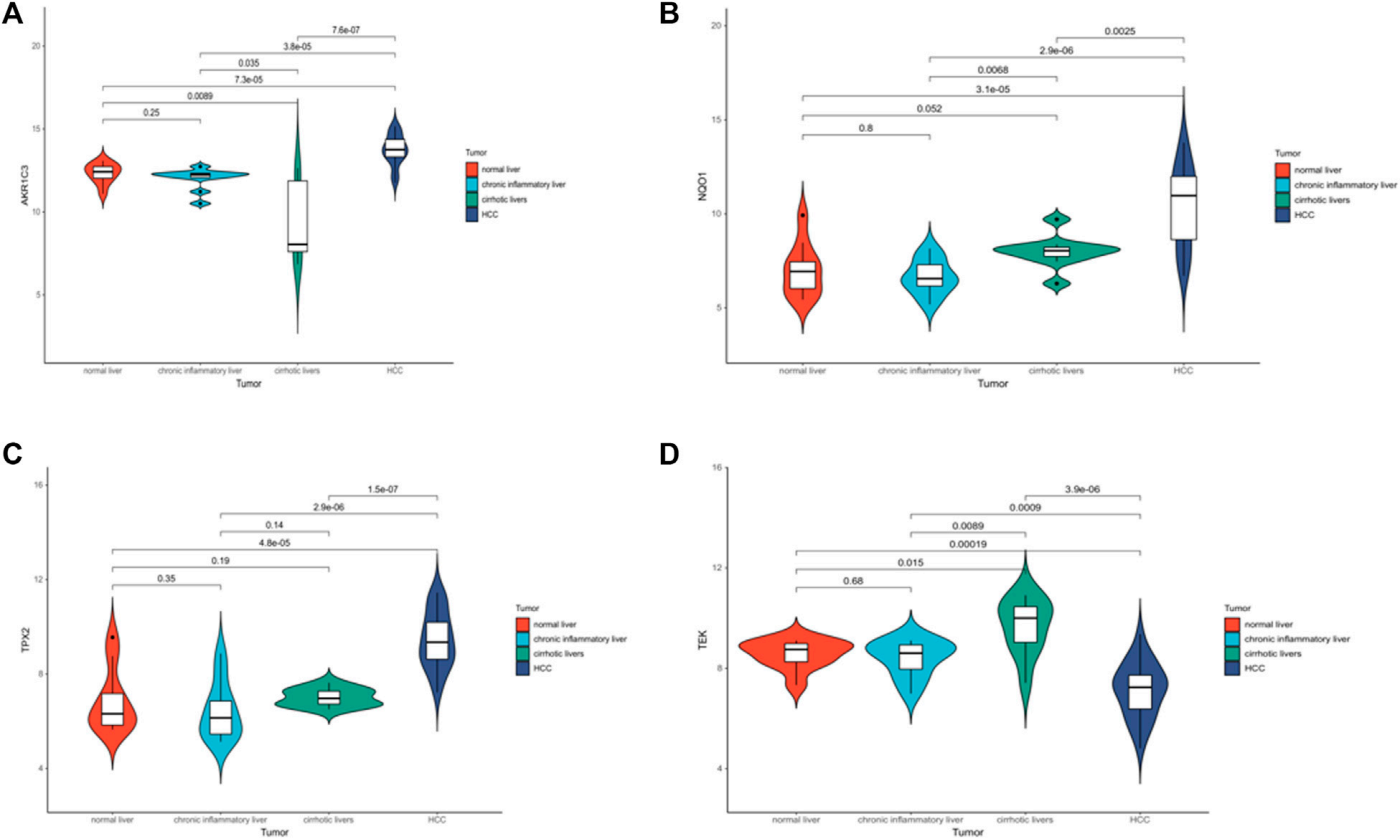
Identification of differentially expressed genes. **(A)** Identification of highly correlated genes between clusters 1 and 2. **(B)** Identification of highly correlated genes between clusters 1 and 3. **(C)** Identification of highly correlated genes between clusters 2 and 3. **(D)** Gene intersection between clusters was screened for overlapping genes.

There were 10 DEGs, including 9 downregulated and 1 upregulated gene (clusters 1 and 2) (Figure 3A). A total of 10 DEGs were found between clusters 1 and 3, including 4 downregulated and 6 upregulated genes between (clusters 2 vs. 3) (Figure 3B). And 16 DEGs, including 4 downregulated and 12 upregulated genes were determined (cluster 1 vs. 2) (Figure 3C). These DEGs were overlapped to determine 4 genes as key diagnostic target genes for subsequent analysis (Figure 3D).

### Key target genes demonstrated significant differential expression between cirrhosis and other group

**FIGURE 4:**
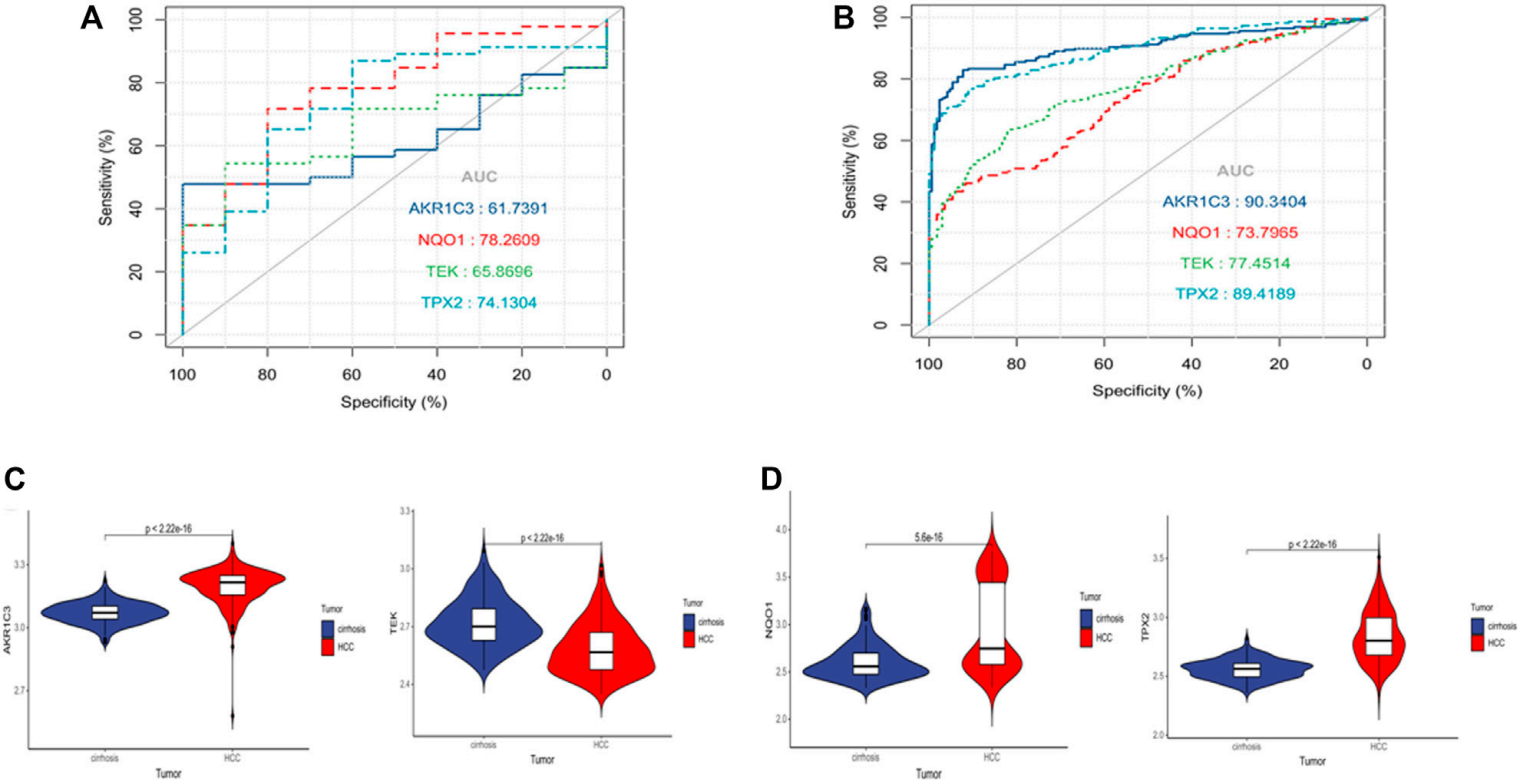
Analysis of the prognostic value of target genes. **(A–D)** Violin plots showing the relative expression of AKR1C3, NQO1, TEK and TPX2 in normal, cirrhotic and HCC.

A violin plot shows that the expression of AKR1C3, NQO1, TEK and TPX2 was significantly associated with different disease states (normal, cirrhosis or liver cancer) (*p* < .05) (Figures 4A–D) (GSE54238). The above results suggest that target genes can be used as independent prognostic factors or combined with existing clinical indicators.

### Evaluation and validation of the diagnostic performances of the target genes on cirrhosis

The AUCs for AKR1C3, NQO1, TEK, and TPX2 were .62, .78, .66, and .74 in the TCGA dataset (Supplementary Figure S1A). These genes also demonstrated good diagnostic performance in the validation dataset GSE63898. The AUCs of expression of AKR1C3, NQO1, TEK, and TPX2 between cirrhosis and HCC were .9, .74, .77, and .89(Supplementary Figure S1B), proving that the four target genes have predictive value. The violin plot with GSE63898 were also consistent with the GSE54238 analysis (Supplementary Figures S1C, D).

### The relationship between target genes and immune infiltration

We tried to determine whether the expression of target genes is related to tumor immune infiltration. We used CIBERSORT to infer the abundance of 22 immune cells. AKR1C3 expression was significantly different among CD4 helper T cells, CD4 memory resting T cells, regulatory T cells (Tregs), and resting NK cells (Supplementary Figure S2A). The expression of NQO1 varied among CD4 memory resting T cells, regulatory T cells (Tregs), resting NK cells, etc. (Supplementary Figure S2B). TEK expression was significantly inconsistent in immune cells such as CD8^+^ T cells and CD4^+^ memory resting T cells (Supplementary Figure S2C). TPX2 differs among cells such as CD4 helper T cells, CD4^+^ memory resting T cells, and regulatory T cells (Tregs) (Supplementary Figure S2D). The target genes obtained by the intersection of the above immune cells were significantly correlated with CD4^+^ memory resting T cells (*p* < .05).

Therefore, we investigated whether drug targets were associated with immune infiltration in HCC from the TIMER database. AKR1C3 expression was significantly correlated with tumor purity (*p* = 4.71e-01) (Supplementary Figure S3A). NQO1 expression was significantly correlated with tumor purity (*p* = 2.25e-03) (Supplementary Figure S3B). TEK expression was significantly correlated with tumor purity (*p* = 4.29e-16) (Supplementary Figure S3C). TPX2 expression was significantly correlated with tumor purity (*p* = 1.23e-03) (Supplementary Figure S3D).

### Knockdown of AKR1C3 and TPX2 suppressed tumor magliance of HCC cells

Considering the important role of AKR1C and TPX2 in carcinogenesis. We knocked down the 2 genes with 2 specific siRNAs of each gene in SNU423 cells, respectively. qRT-PCR and western blot was performed to evaluate the efficiency of siRNAs (Supplementary Figures S4A, S5B). Knockdown of AKR1C3 and TPX2 significantly suppressed cell proliferation (Supplementary Figure S4C). Woundhealing assay revealed that knockdown of AKR1C3 suppressed cell migration significantly (Supplementary Figure S5A), and knockdown of TPX2 also slightly suppressed cell migration (Supplementary Figure S5B). Trans-well assay was performed to evaluate invasive ability of HCC cells. As shown in Supplementary Figures S5C, D, knockdown of either AKR1C3 or TPX2 suppressed invasive ability of SNU423 cell significantly.

## Discussion

The liver is the major organ that metabolizes three major nutrients: protein, fat, and carbohydrate (Moriwaki et al., 2004; Charlton, 2006). The gut-liver axis is essential in liver fibrosis and cirrhosis (Bajaj, 2019). It is a complex and regulated process that balances substrate production and degradation (Qin et al., 2014). Identifying diagnostic therapeutic biomarkers may help clinicians improve treatment strategies (Acharya and Bajaj, 2019). Therefore, it is necessary to evaluate the key drug targets in liver cirrhosis.

We use the bioinformatical analysis to verify that AKR1C3, NQO1, TEK, and TPX2 have good diagnostic performance in patients with cirrhosis. These genes may serve as key biomarkers for diagnosing patients with cirrhosis. We further explored the impact of AKR1C3 or TPX2 on the development of liver cancer *in vitro*. We found that knockouts of AKR1C3 or TPX2 can significantly inhibit the invasion of liver cancer cells. AKR1C isotic enzyme 2 and 3 may work in male-related liver diseases such as cirrhosis (White et al., 2014). Studies have proved that STAT3 and NF-κB inhibitors have caused liver star apoptosis and accelerated live fibrosis recovery (Sommerhalder et al., 2021). NF-κB and STAT3 activate the proliferation and metastasis of hepatoma carcinoma cells (He and Karin, 2011). Knockout of TPX2 in the hepatoma carcinoma cell system can reduce cell growth and induce apoptosis by blocking G2/m. Activating PI3K/AKT signaling pathway can promote the production of internal blood vessels, aggravating the process of liver cirrhosis (Wang et al., 2019). TPX2 can promote the development of HCC by activating the PI3K/AKT pathway (Zeng et al., 2020; Huang et al., 2019). The expression of NQO1 is related to HCC’s internal liver recurrence and poor prognosis (Shimokawa et al., 2020). TEK delays tumor growth, slows down metastasis, and enhances the response to accompanying cytotoxic therapy (Goel et al., 2013). Verifying our research results can provide new ideas for the progress of cirrhosis.

Furthermore, immune infiltration analysis shows a correlation between target genes and several immune cells (Tian et al., 2022). We revealed that 4 target genes are significantly related to CD4^+^ memory static T cells. CD4^+^ memory static T cells secrete iconic cytokines IL-4, IL-10, and IFN-G, and stimulate other immune cells such as NK cells, to media the progress of liver fibrosis (Liu et al., 2022). The CD4^+^ T cells and CD4/CD8 ratio in liver cirrhosis is reduced. CD4^+^ T cells, including CD4^+^ effects T cells and CD4^+^ memory T cells increase (Bárcena et al., 2019). The change in the target gene reflects the liver fibrosis immune microenvironment changes. Therefore, we investigated whether the drug target is related to the immunohistos in HCC. The expression of the 4 target genes is significantly related to the purity of the tumor. Compared with normal liver cells, CD4^+^ memory static T cells in HCC increased significantly (Viveiros et al., 2019). Emerging cancer cells can be identified and killed by many immune cells in tumor treatment, such as CD8^+^ and CD4^+^ memory static T cells (Mukaida and Nakamoto, 2018). For instance, CD4^+^ memory static T cells inhibit the development of liver cancer and media for tumor retreat (Ma et al., 2016). The increase in cytotoxic CD4^+^ T cells is related to disease-free survival and total survival (Fu et al., 2013). Our results are consistent with these reports and verify the correlation between immune cells and target genes.

The development of cirrhosis is an essential clinical marker in patients with chronic liver disease. It predicts an increased risk of morbidity and a decreased probability of survival. Therefore, a comprehensive understanding of the prognostic character model of liver cirrhosis is critical to improve clinical outcomes. In addition, further studies on the interactions between drug targets and immune cells can elucidate the pathogenesis of liver cirrhosis and provide new opportunities for immunotherapy.

## Conclusion

We explored the diagnostic key targets of Tanshinone IIA in liver cirrhosis through exploaration of comprehensive dataset including health, liver cirrhosis and liver cancer patients. The diagnostic performance of target genes was assessed and further verified in the external dataset. We found that the 4 key drug targets could be used as effective diagnostic biomarkers. And the knockouts of AKR1C3 or TPX2 can significantly inhibit the invasion of liver cancer cells.

## Data Availability

The datasets presented in this study can be found in online repositories. The names of the repository/repositories and accession number(s) can be found in the article/Supplementary Material.

## Supplementary material

SUPPLEMENTARY FIGURE S1 Evaluation and validation of target genes for diagnosis of cirrhosis. **(A)** ROC analysis of key target genes in GSE54238. **(B)** ROC curve of the key target genes in GSE63898. **(C)** Differential expression of key target genes in cirrhosis and HCC group in GSE63898.

SUPPLEMENTARY FIGURE S2 Immune infiltration relationship between target genes and 22 immune cell subsets. **(A)** Differences in immune cell distribution between high and low AKR1C3 expression groups and low. **(B)** Differences in immune cell distribution between high and low NQO1 expression groups and low. **(C)** Differences in immune cell distribution between high and low TEK expression groups and low. **(D)** Differences in immune cell distribution between high and low TPX2 expression groups and low.

SUPPLEMENTARY FIGURE S3 Correlation of target gene expression with TME. **(A)** AKR1C3 expression was significantly correlated with tumor purity. **(B)** NQO1 expression was significantly correlated with tumor purity. **(C)** TEK expression significantly correlated with tumor purity. **(D)** TPX2 expression significantly correlated with tumor purity.

SUPPLEMENTARY FIGURE S4 mRNA and protein expression of AKR1C3 and TPX2 **(A)** qRT-PCR of AKR1C3 and TPX2 in SNU423 cells after transfecting with specific siRNAs. **(B)** Western blot of AKR1C3 and TPX2 after knockdown of the specific gene. **(C)** Cell numbers of the cells on 72 h post-transfection with siRNA.

SUPPLEMENTARY FIGURE S5 Knockdown of AKR1C3 and TPX2 suppressed tomor magliance of HCC cells. **(A)** Left panel: wound healing assay of cells after transfecting with AKR1C3 siRNAs. Right panel: statistical anaylsis results of migration diatance percentage, 5 measurements was calculated for each group. **(B)** Left panel: wound healing assay of cells after transfecting with TPX2 siRNAs. Right panel: statistical anaylsis results of migration distance percentage, scale bar: 400 μm. **(C)** Trans-well assay of cells after knockdown of AKR1C3 or TPC2. **(D)** Statistical analysis of invasive cells, data was shown as percentage. Five views was calculated for each group. Each experiment has been repeated for at least 3 times. *, *p* < .05; **, *p* < .01.
