# Supplementary figures and images for: Integrated bioinformatical and in vitro study on drug targets for liver cirrhosis based on unsupervised consensus clustering and immune cell infiltration
Source: Front Pharmacol. 2023 Jan 4;13:909668. doi: 10.3389/fphar.2022.909668 (PMC9846563; doi:10.3389/fphar.2022.909668)

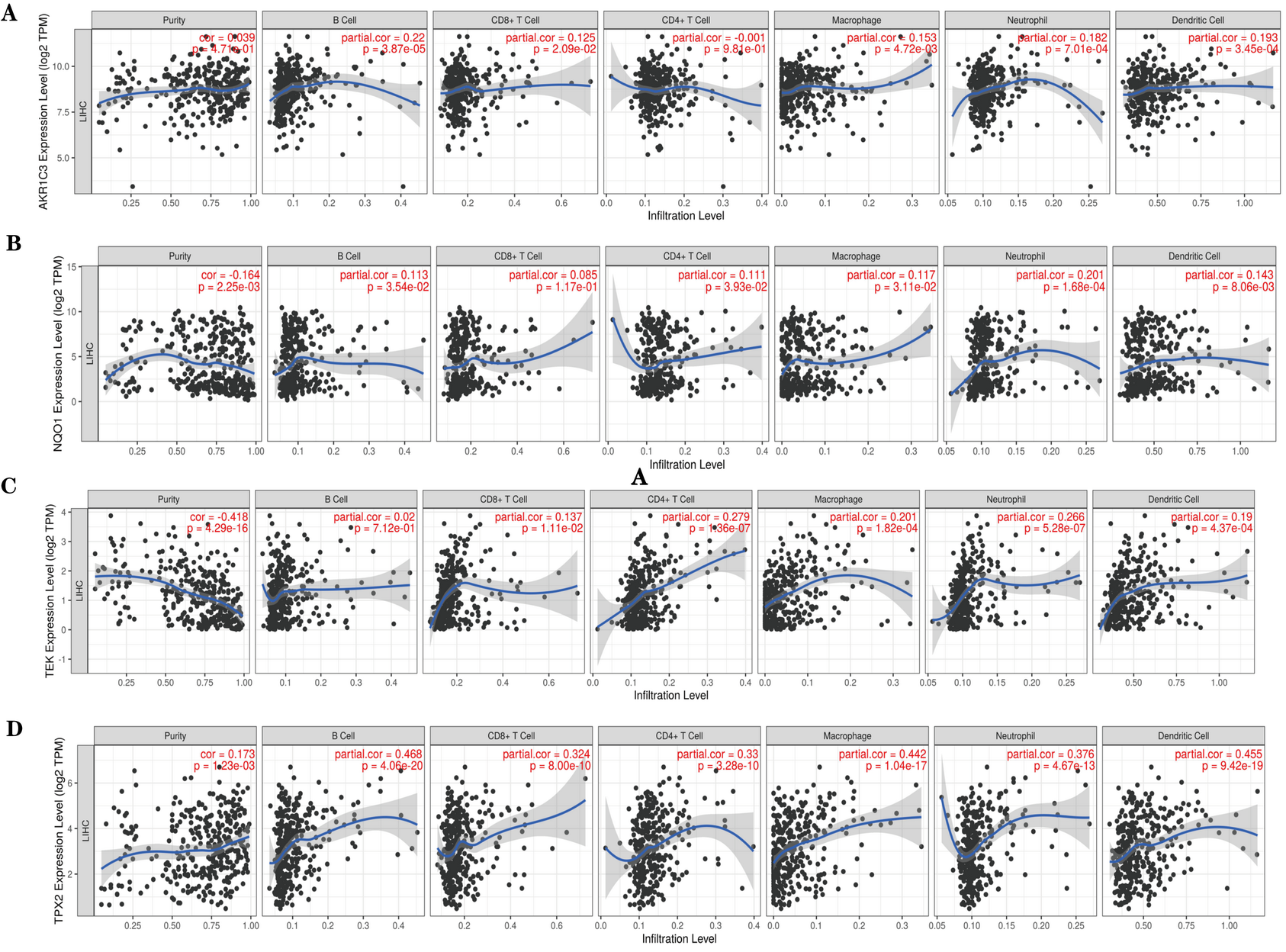

Supplement: Supplementary file 1 [file Image3.TIF]

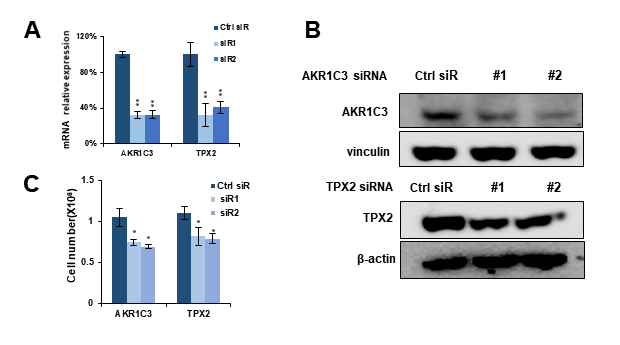

Supplement: Supplementary file 2 [file Image4.tif]

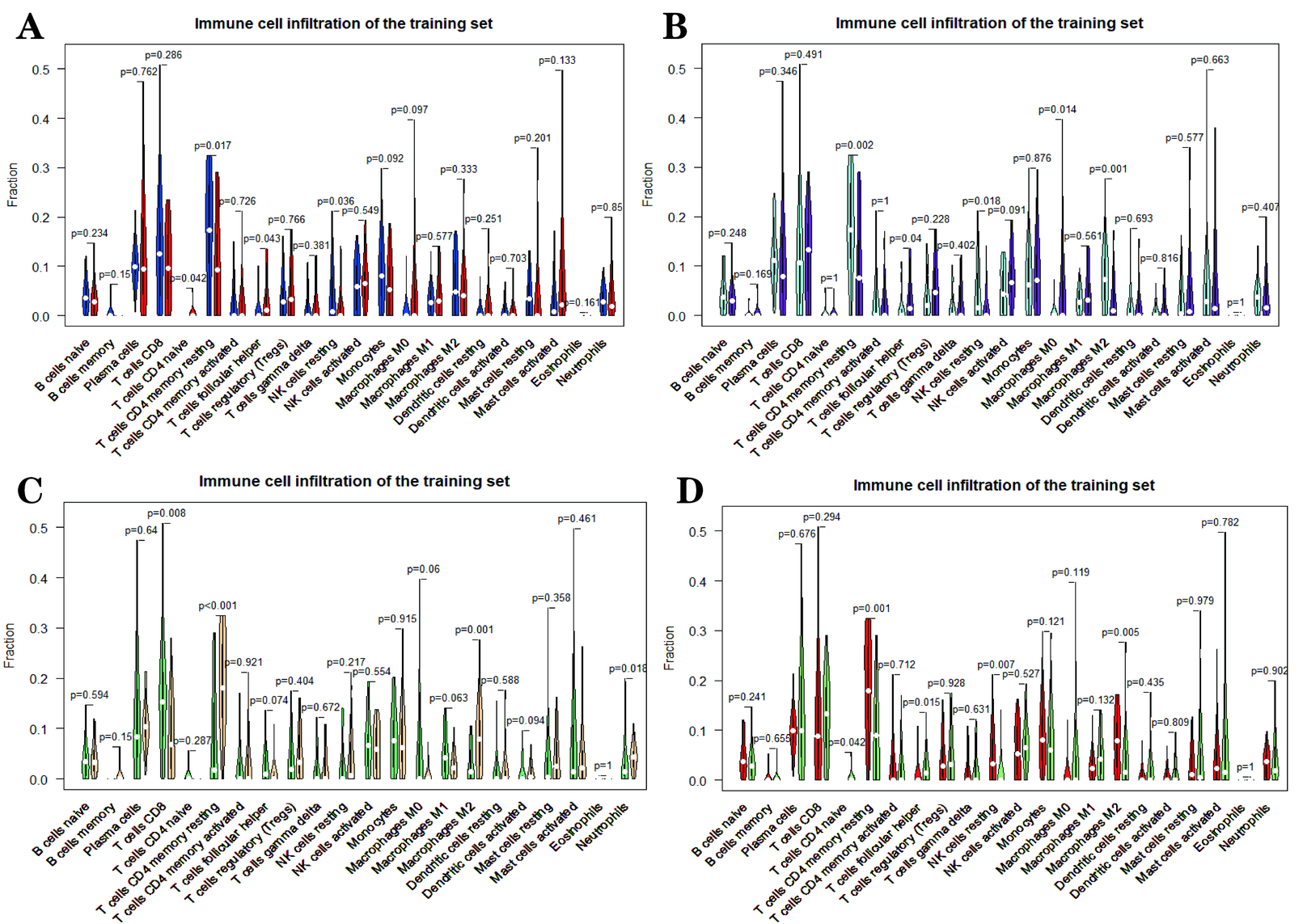

Supplement: Supplementary file 3 [file Image2.TIF]

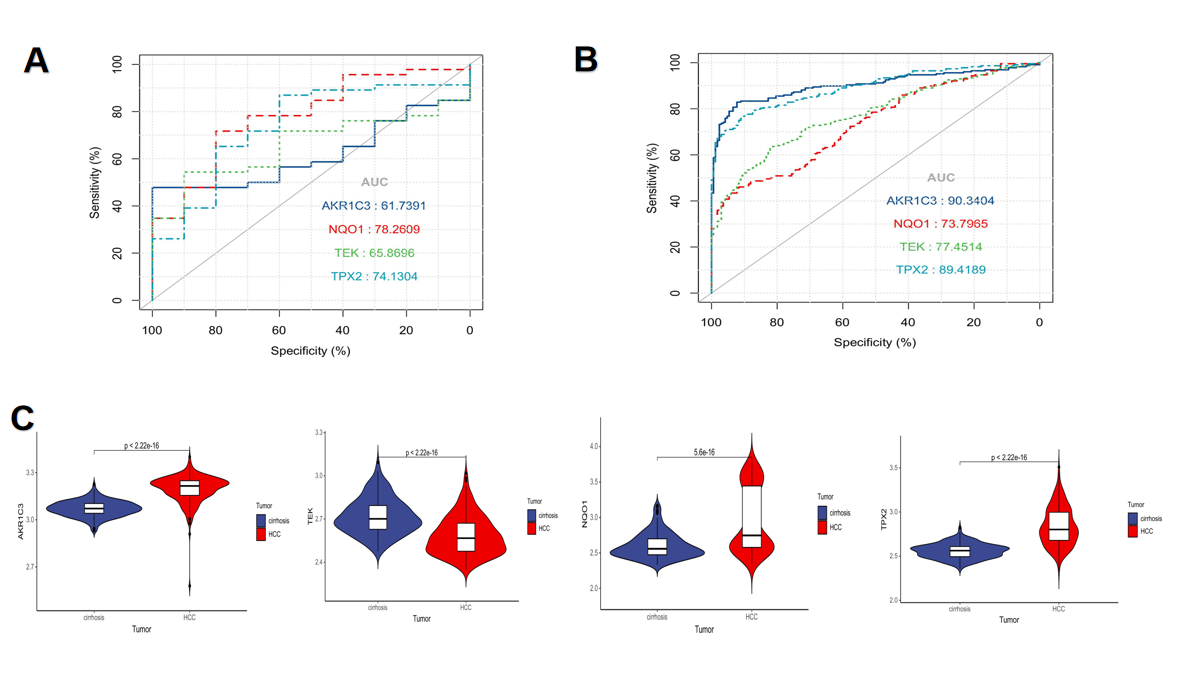

Supplement: Supplementary file 4 [file Image1.TIF]

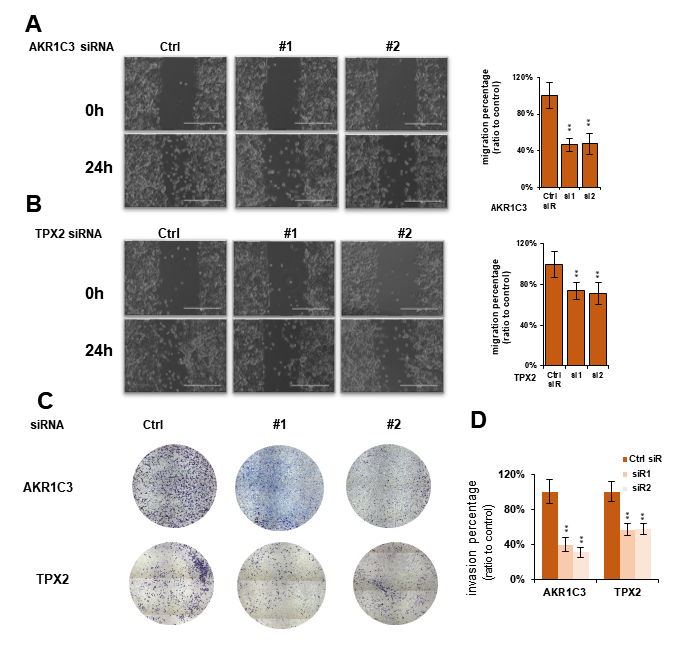

Supplement: Supplementary file 5 [file Image5.tif]
